# Meeting the Challenge of Influenza Pandemic Preparedness in Developing Countries 

**DOI:** 10.3201/eid1503.080857

**Published:** 2009-03

**Authors:** David S. Fedson

**Affiliations:** University of Virginia, Charlottesville, Virginia, USA (retired)

**Keywords:** Pandemic, influenza, developing countries, H5N1, generic agents, statins, PPAR agonists, World Health Organization, perspective

## Abstract

Developing countries face unique difficulties preparing for an influenza pandemic. Our current top-down approach will not provide these countries with adequate supplies of vaccines and antiviral agents. Consequently, they will have to use a bottom-up approach based on inexpensive generic agents that either modify the host response to influenza virus or act as antiviral agents. Several of these agents have shown promise, and many are currently produced in developing countries. Investigators must primarily identify agents for managing infection in populations and not simply seek explanations for how they work. They must determine in which countries these agents are produced and define patterns of distribution and costs. Because prepandemic research cannot establish whether these agents will be effective in a pandemic, randomized controlled trials must begin immediately after a new pandemic virus has emerged. Without this research, industrialized and developing countries could face an unprecedented health crisis.

## Abstract 2

One-sentence summary for table of contents: These countries should consider using inexpensive generic agents to confront the next pandemic.

More than a decade ago, the first human cases of disease caused by avian influenza virus A (H5N1) appeared in Hong Kong Special Administrative Region, People’s Republic of China. Five years ago, influenza virus A (H5N1) reemerged to cause highly lethal human disease in Southeast Asia. Health officials are concerned that these cases could be the harbinger of the next influenza pandemic. As a result, virtually all industrialized countries and many developing countries have mounted extensive pandemic preparedness efforts. However, as pointed out recently by Oshitani et al., industrialized countries face “unique and difficult issues, which make preparing for a pandemic more challenging” (*1*).

## Why a Top-Down Approach to Confronting the Next Pandemic Will Not Work

If a pandemic form of influenza virus A (H5N1) emerges within the next few years, all countries will have to depend almost entirely on egg-derived inactivated adjuvanted influenza vaccines. For developing countries, this approach will not succeed. Estimates show that within the first 6–9 months of a pandemic outbreak, vaccine companies will be only able to produce enough doses to vaccinate ≈700 million persons (*2*). This number is less than the combined populations of the 9 countries that produce almost all of the world’s seasonal influenza vaccines. These countries will first use their vaccines to ensure that their own populations are protected. Non–vaccine-producing countries, both industrialized and developing, will have to wait.

In 2005, a representative of the World Health Organization (WHO) Global Programme on Influenza concluded that “most developing countries will have no access to a vaccine during the first wave of a pandemic and perhaps throughout its duration” (*2*). Since then, WHO has worked to build a stockpile of ≈1500 million doses of vaccine against influenza virus A (H5N1) for developing countries (*3*), and 2 companies have pledged to provide WHO with 110 million doses. In 2007, a WHO scientific consultation on how to use this stockpile concluded, “If there is sufficient early warning that an outbreak of influenza (H5N1) is due to a virus that is capable of sustained human-to-human transmission, then theoretically there may be a relatively limited ‘window of opportunity’ to stop the spread of the virus before it spreads nationally or internationally. … However, a containment effort would be feasible only in settings where the number of localized cases are [sic] still limited, where adequate logistical support is available, and where the national government is supported by international assistance” (*3*). The vaccine stockpile on which these efforts would depend does not yet exist.

Several industrialized countries are stockpiling vaccines against influenza virus A (H5N1) that might be used for prepandemic vaccination, but Oshitani et al. note that “both pandemic and prepandemic vaccines would not be available in developing countries unless an international mechanism exists to share such vaccine with them at low cost” (*1*). Even if limited supplies of vaccines could be produced for developing countries, no international mechanism is in place to pay for and distribute the vaccines, and WHO has yet to announce plans to set one up. Thus, when the next pandemic virus emerges, almost no vaccines will be available in developing countries to slow its spread (*1**,**2*).

Because global supplies of vaccines against pandemic viruses will be limited, government officials in a few industrialized countries have placed their hopes on stockpiles of antiviral agents, primarily oseltamivir, an expensive neuraminidase inhibitor. In 2005, WHO established its Southeast Asian Influenza Clinical Research Network to study neuraminidase inhibitor treatment of patients infected with viruses that possess pandemic potential (*4*). However, influenza virus A (H1N1) has developed resistance to oseltamivir, and similar antiviral resistance could develop in a future pandemic virus. Five million treatment courses (10 doses per patient) of oseltamivir have been donated to a WHO stockpile, but WHO has no plans to dramatically increase the size of this stockpile. On their own, the governments of a few countries that do not produce influenza vaccines or antiviral agents have purchased supplies of oseltamivir, but their stockpiles are sufficient to treat only 1% of their combined populations (D.S. Fedson, unpub. data). Not surprisingly, developing countries themselves “will not allocate scarce resources to stockpile significant quantities of oseltamivir for an unpredictable influenza pandemic” (*1*). Clearly, the limited supplies of antiviral agents available to developing countries where these infections now occur will scarcely have any effect on a pandemic after it starts to spread.

Influenza virologists report that recent isolates of highly pathogenic influenza viruses (H5N1 and H7N1) have acquired molecular characteristics suggesting they might become more easily transmissible among humans (*5**,**6*). In Indonesia, physicians have reported that everyone infected with the clade 2 influenza virus A (H5N1) who did not receive antiviral treatment has died (Table 1) (*7*). Given extremely limited global supplies of antiviral agents, this is a terrifying observation. If a pandemic virus were to emerge with a level of virulence approaching that of influenza virus A (H5N1) in Indonesia, it could lead to a global population collapse. Many influenza virologists doubt this will ever happen and believe instead that influenza virus (H7N7) or reemergent influenza virus (H2N2) could also cause the next pandemic. Chances are they might be right. Moreover, health officials in national governments and international agencies estimate that expected pandemic deaths will be no more than what can be extrapolated from the 1918–1920 pandemic (*8*). These officials seldom, if ever, use the phrases “population collapse” or “population die off,” and their estimates may also be right. Nonetheless, in a seminal experiment reported in 1974, Webster and Campbell showed that genetic reassortment, the process that gave rise to pandemic viruses in 1957 and 1968, could give rise to a readily transmissible virus of extraordinary virulence (Figure) (9). This experiment and human experience with influenza virus A (H5N1) in Indonesia suggest it would be prudent for all countries to plan for something much worse than what occurred in 1918–1920.

**Table 1:** Relationship between time of onset of antiviral treatment and case-fatality rate in persons with avian influenza A (H5N1) disease in Indonesia, 2003–2007*

Interval between onset of illness and treatment	No. cases	No. deaths	Case-fatality rate, %
<24 h	2	0	0
0–4 d	11	5	45
0–6 d	37	24	65
>6 d	49	40	82
Any treatment	86	64	74
No treatment	33	33	100
All cases	119	97	82

*Adapted from (*7*).

**Figure:**
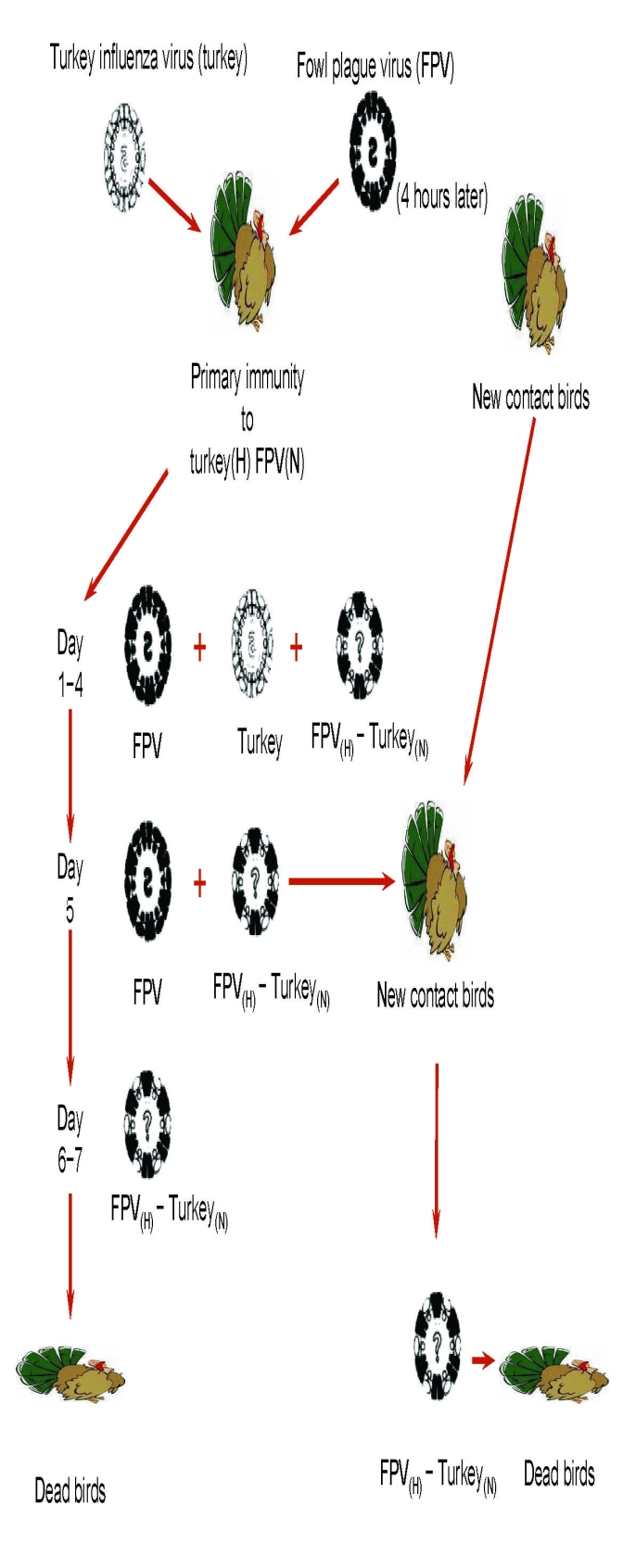
Genetic reassortment and genesis of a new pandemic influenza virus. This study was designed to determine whether the selection and transmission of a new reassortant influenza A virus could occur under experimental conditions in vivo that mimic what might occur in nature. Reassortment between 2 antigenically distinct influenza A viruses was studied in turkeys that had been previously immunized to induce low levels of antibodies to the hemagglutinin (H) of a nonlethal turkey influenza virus (Turkey), and to the neuraminidase (N) of a fowl plague virus (FPV), an avian virus that is highly pathogenic for chickens. Twenty-eight days after immunization, the immunized turkeys were sequentially infected, first with the Turkey virus and 4 h later with FPV. During the first few days, both parent viruses were isolated from the infected turkeys, but by day 4 a reassortant virus containing the FPV hemagglutinin and the Turkey neuraminidase (FPV_(H)_–Turkey_(N)_) was also isolated; within 2 days it became the dominant virus. All infected turkeys died, and only the FPV_(H)_–Turkey_(N)_ reassortant virus could be recovered. In a separate experiment, similarly immunized turkeys were again sequentially infected, but on day 5 a group of nonimmunized or selectively immunized turkeys (Turkey_(H)_ FPV_(N)_) were placed in the same room. All contact birds soon died of fulminant infection caused by the FPV_(H)_–Turkey_(N)_ reassortant virus. These experiments demonstrated that under conditions of selective primary immunity, a new virus could be generated through genetic reassortment in vivo and that this reassortant virus could be readily transmitted to contacts. The reassortant virus caused uniformly fatal disease in primary infected and contact birds. Thus, under the conditions of these experiments, genetic reassortment gave rise to a new influenza virus that led to a total population collapse. Adapted from Webster and Campbell (*9*).

The current approach to pandemic planning for all countries involves small groups of health officials, influenza scientists, and company executives, most of whom come from industrialized countries. For the foreseeable future, this top-down approach will be incapable of providing developing countries with timely supplies of affordable vaccines and antiviral agents. (Most industrialized countries that do not produce influenza vaccines will have similar difficulties, at least for the first pandemic wave.) The Indonesian Health Minister, for one, understands this. With little prospect that people in her country will be able to obtain vaccines against pandemic viruses, she precipitated a standoff with WHO by announcing in February 2007 that unless Indonesia is able to gain access to supplies of vaccines against pandemic viruses, her country will no longer share its influenza viruses A (H5N1) with WHO’s laboratory-based surveillance system (*2*). Despite unorthodox arguments (*10*), her position has garnered wide support from the health ministers of many developing countries (*11*). Recently, Indonesia agreed to share influenza virus A (H5N1) sequences (not the viruses themselves) with the Global Initiative on Sharing Avian Influenza Data, but the country no longer promptly reports deaths from influenza virus A (H5N1), in defiance of new International Health Regulations. WHO has been unable to come up with a solution to this impasse.

In identifying the major issues and challenges of a pandemic threat facing developing countries, Oshitani et al. have called for better preparedness planning, improved systems for medical care and public health, expanded use of nonpharmaceutical interventions, and strengthened core capacities for seasonal influenza surveillance and vaccination (*1*). They recognize that this is a challenge few developing countries will be able to meet, but go on to say, “Preparing for a pandemic by simply strengthening preparedness within a single country is not possible. A pandemic is a global issue, and pandemic preparedness should be considered from a global perspective” (*1*). In practical terms, what exactly does this mean? The record thus far indicates that truly international efforts to prepare for pandemic vaccination and antiviral use have been meager. In almost all instances, these efforts have been vastly outweighed by efforts that reflect national concerns and interests.

## A Bottom-Up Approach that Developing Countries Can Use to Confront the Next Pandemic

A top-down approach will not ensure that adequate and affordable supplies of vaccines against pandemic viruses and antiviral agents can be produced and distributed in time to protect populations in developing countries. Transferring technology for vaccine and antiviral agent production to a small number of developing countries will proceed slowly and will inevitably fail to meet the needs of neighboring countries not favored by these programs (*12*). Consequently, developing countries must consider an alternative bottom-up approach to pandemic control, an approach based on existing healthcare workers and institutions and that uses inexpensive and widely available generic agents that have intrinsic antiviral activities or that modify the host response (*13**,**14*).

Many influenza scientists doubt this approach will work (*14**–**16*). Nonetheless, as reviewed elsewhere (*13**,**14*), several retrospective studies suggest that outpatient statins (drugs taken to lower cholesterol levels and prevent cardiovascular diseases) reduce 30-day pneumonia mortality rates by ≈50% (Table 2) (*17**–**22*). Most investigators agree that these observational studies must be interpreted with caution and that promising results should be followed by prospective clinical trials. One such trial is already under way, and a preliminary report has shown that in 67 pneumonia patients in intensive care units, treatment with statins reduced the hospital mortality rate by 51% (p = 0.026) (*23*). Pulmonary investigators also believe that peroxisome proliferator–activated receptor (PPAR) α and PPARγ agonists (fibrates and glitazones, respectively) could be used to treat acute lung injury (*14*). An important experimental study has shown that the fibrate gemfibrozil, a PPARα agonist used to prevent heart disease, reduced mortality rates in mice infected with influenza virus (H2N2) by 54% (*24*). Statins and PPAR agonists have antiinflammatory and immunomodulatory activities, and there is considerable molecular cross-talk between these agents (*14*). Moreover, combination treatment is safe, and in patients with cardiovascular diseases, clinical benefits are additive. Used either alone or together, this treatment might similarly benefit patients during an influenza pandemic.

**Table 2:** Recent studies of patients with pneumonia treated with statins*

Investigator (reference)	Study design and population	Principal outcome	Adjusted odds ratio (95% CI) or % reduction (p value)
van der Garde et al. (*17*)	Case–control diabetes patients, 4,719/15,322	Pneumonia hospitalization	0.50 (0.28-0.89)
Schlienger et al. (*18*)	Case–control, 1,227/4,734	Pneumonia hospitalization	0.63 (0.46–0.88)
		30-day pneumonia mortality rate	0.47 (0.25–0.88)
Mortensen et al. (*19*)	Retrospective cohort, 1,566/7,086	30-day pneumonia mortality rate	0.54 (0.42–0.70)
Chalmers et al. (*20*)	Prospective cohort, 257/750	30-day pneumonia mortality rate	0.46 (0.25– 0.85)
Thomsen et al. (21)	Retrospective cohort, 1,372/28,528	30-day pneumonia mortality rate	0.69 (0.58–0.82)
Majumdar et al. (*22*)	Prospective cohort, 325/3,090	Hospital mortality rate and ICU admission (adjusted for administrative data)	0.88 (0.63–1.22)
		Hospital mortality rate and ICU admission (adjusted for age, propensity score, clinical data, and functional status)	1.10 (0.76–1.60)
Choi et al. (*23*)	Randomized controlled trial, ICU treatment; 33 with atorvastatin and 34 controls	ICU mortality rate	45.4 (0.08)
		Hospital mortality rate	51.2 (0.026)

*Except for the inpatient randomized controlled trial of Choi et al (*23*), recent treatment in the observational studies was defined as a statin prescription within a period of 30 days (*18*) to 90 days before hospitalization for pneumonia. CI, confidence interval; ICU, intensive care unit.

Other generic agents, some with direct activity against influenza virus, should also be considered (*14*). Chloroquine, long used as an antimalarial drug, increases endosomal pH and acts as an antiviral agent by impairing virus release into the cytosol. Resveratrol, a polyphenol found in red wine, reduces influenza mortality rates in experimentally infected mice (*25*). Catechins (found in green tea) (*26*) and curcumin (turmeric; found in curry powder) (*27*) have numerous cell-signaling effects, suggesting that they too might be beneficial. A combination of agents that act on both the host response and the virus might be required.

It is becoming increasingly difficult for investigators to ignore arguments for treating the host response to influenza. Recently, investigators showed that giving a neuraminidase inhibitor to mice infected with influenza virus A (H5N1) was not nearly as effective as treating the mice with an antiviral agent and 2 immunomodulatory agents, mesalazine, a PPARγ agonist, and celecoxib, a cyclooxygenase (COX)–2 inhibitor (*28**,**29*). In this model, targeting the host response to infection was essential for improving survival rates and times. More important, 2 studies in mice showed that intratracheal administration of either a fragment of the PB1-F2 protein of the 1918 influenza virus (*30*) or an inactivated influenza virus A (H5N1) (*31*) caused severe acute lung injury similar to that seen in fatal human cases of influenza (either from the 1918–1920 pandemic or from the current H5N1 subtype). In these experimental models, there was no virus replication. Thus, antiviral agents would have had no effect. Although we still lack direct evidence that one or more antiinflammatory and immunomodulatory agents alone would effectively treat human influenza virus A (H5N1) infections, these results and those from the study of influenza virus (H2N2)–infected mice treated with gemfibrozil (*24*) suggest these agents might be effective.

What makes these agents so important is that many of them are currently being produced as generic drugs in developing countries (*13**,**14*). These drugs are inexpensive, could be produced in abundance, and could even be stockpiled and made available for use on the first day of a pandemic. No matter what is accomplished in the years ahead, adequate supplies of vaccines and specific antiviral agents will never be available to persons in developing countries on the first pandemic day.

## A Research Agenda to Establish a Generic Approach to Pandemic Treatment and Prophylaxis

What types of research on generic agents do we need before the pandemic virus appears? First, experimental studies of several candidate treatment regimens must be undertaken in mice infected with influenza virus A (H5N1) or 1918-like viruses (Table 3). The agents used in these studies might have antiinflammatory and immunomodulatory or antiviral properties (some might have both), but all must be generic agents that are currently produced in developing countries. Admittedly, these experimental studies in mice will have limitations (*32*), but they should identify avenues for further research. Once a few treatment regimens have been shown to be effective in mice, they should be tested in ferrets. Later, 2 or 3 of the most promising regimens should be tested in nonhuman primates.

**Table 3:** Research agenda to establish whether generic agents could be used for treatment and prophylaxis of a pandemic caused by a subtype H5N1-like influenza

1. Test candidate treatment regimens in mice, ferrets, and nonhuman primates to identify specific generic agents that might be effective in managing a pandemic
2. Study promising generic treatments in cell culture and animals to define the molecular mechanisms that explain their beneficial effects against influenza virus A (H5N1) and 1918-like influenza viruses
3. Conduct a global analysis to identify developing countries where these generic agents are produced and determine quantities produced, surge capacities, patterns of distribution, and costs to public programs
4. Establish an international process to coordinate or manage the stockpiling of generic agents and/or their distribution once a pandemic virus has emerged
5. Plan to conduct randomized controlled trials of promising generic treatments immediately after the emergence of a new pandemic virus


After demonstrating the effectiveness of 1 or more treatment regimens in animals, influenza virologists should then use in vitro systems to define the molecular mechanisms responsible for their protective activity. However, some of these agents will have broader effects on the host response. For example, although administering a COX-2 inhibitor along with a PPARγ agonist improved survival rates and times in mice infected with influenza virus A (H5N1) (*21*), another study showed that selective COX-2 inhibition was detrimental to the resolution of acute lung injury (*33*). Most influenza scientists focus their research on the virus or on cell-signaling events associated with viral pathogenesis (*34*). Yet the pathophysiologic effects of severe infections involve the entire host, something well known to researchers who study sepsis (*35**–**37*). Their studies have shown that statins and PPAR agonists stabilize myocardial and microvascular function, preserve integrity of pulmonary endothelial cell tight junctions and prevent pulmonary edema, and promote resolution of acute inflammation (*13**,**14*). Thus, other investigators with laboratory and clinical expertise in critical care, cardiopulmonary diseases, and endocrinology and metabolism must be recruited to explore in animals the molecular mechanisms underlying these broad treatment effects on the host. However, in undertaking this research, investigators must not forget that their primary goal is to find effective ways to manage a pandemic in populations and not simply to explain in more precise terms the harmful effects of pandemic virus infection in individuals.

While these studies are under way, an analysis should be undertaken for each candidate agent to determine which companies produce them, where each is manufactured, annual levels of production (and surge capacity), patterns of distribution to other developing countries, and costs for public markets (Table 3). Special attention must be given to companies that follow Good Manufacturing Practices to minimize the risk that some of these agents might be counterfeit. When animal studies have defined 1 or more promising regimens, an international process must be set up to develop logistics for financing, producing, and distributing each agent.

Where feasible, clinical trials of promising treatment regimens might be undertaken in patients with severe seasonal influenza. In a few instances, clinicians might choose to treat patients infected with influenza virus A (H5N1) on a compassionate basis (*12*). However, none of these limited studies will guarantee that promising treatments in the prepandemic period will be effective against a true pandemic virus. Thus, careful plans must be made during the prepandemic period that will enable investigators to conduct randomized controlled trials of promising generic regimens during the early weeks of a new pandemic. If the case-fatality rate is similar to that of influenza virus A (H5N1) (≈60%), trials will not need to be large (Table 4). Within a few days, investigators should be able to recruit sufficient numbers of patients to satisfy statistical requirements.

**Table 4:** Sample size requirements for a randomized controlled trial of treatment to reduce deaths in a pandemic caused by a subtype H5N1-like influenza*

Case-fatality rate, %		Reduction in no. deaths, %	Total sample size (power)		
Untreated	Treated		80%	90%	95%
50	37.5	25	530	690	850
50	25	50	140	170	210
50	12.5	75	60	80	90

*1:1 randomization of persons to the 2 treatment groups, α = 0.05 (2-sided), χ^2^ test (continuity corrected). The example shown assumes a case-fatality rate of 50%, which is similar to what has been seen for patients infected with influenza virus A (H5N1). If a new pandemic virus is associated with a lower case-fatality rate, sample sizes required to show similar reductions in case-fatality rates would have to be larger.

Planning for clinical trials during the prepandemic period must start with identifying clinical investigators who will conduct these trials and institutions that will sponsor their work. Supplies of the agents to be tested must be set aside, study protocols written, and ethical approval obtained. A mechanism for rapid regulatory approval must be developed to enable trials to be conducted wherever the pandemic virus first emerges. A financing mechanism must be established that enables immediate access to funds necessary to support the trials. Finally, an internet-based communication strategy must be devised that ensures prompt dissemination of study results to physicians and health officials worldwide.

None of this research on generic agents will be possible without international coordination. Thus far, the top-down approach that has characterized vaccine and antiviral research and development has lacked an international system for coordination and management to ensure rapid progress (*2*). Likewise, nothing has been done to ensure worldwide production and distribution of the vaccines and antiviral agents being developed. A similar approach must not be allowed to govern the research agenda for generic agents.

Experience with the severe acute respiratory syndrome (SARS) in 2003 shows us how we could do much better. When SARS first came to international attention, WHO quickly established 3 virtual networks of experienced virologists, clinicians, and epidemiologists (*38*). By sharing experiences and findings on secure websites and in daily teleconferences, investigators soon identified and sequenced the SARS coronavirus, defined the clinical features of the disease, and established practical measures for clinical management and epidemiologic control. Surprisingly, WHO has not set up a similar system to coordinate research and development of vaccines against pandemic viruses and antiviral agents, despite the far greater threat to global health inherent in an influenza (H5N1) pandemic (*2*). Given escalating pressure from developing countries, WHO can ill afford to adopt the same slow approach to establishing the scientific basis for using inexpensive and widely available generic agents for pandemic control.

## Conclusions

Oshitani et al. correctly emphasize that preparing for the next pandemic requires a global perspective, but this does not necessarily mean that the measures used to confront the pandemic in developing countries must be supplied through an internationally organized top-down process. An international process will surely be required for distributing vaccines and antiviral agents, but experience indicates that the process will be slow and cumbersome and supplies of these agents will remain scarce (*2*). Nonetheless, developing countries will need abundant supplies of effective agents, and abundance will be guaranteed only if these agents are generic, inexpensive, and produced in developing countries themselves.

It is too soon to know whether generic agents could be used to confront the next influenza pandemic, yet developing countries lack realistic alternatives. For this reason, their leaders must convince scientists and international organizations, including WHO, of the urgent need for research to determine whether these inexpensive agents could mitigate the effects of a pandemic. Otherwise, developing and industrialized countries alike could be faced with an unprecedented global health crisis.
